# From Infection to Autoimmunity: *S. pyogenes* as a Model Pathogen

**DOI:** 10.3390/microorganisms13061398

**Published:** 2025-06-16

**Authors:** Virginia Girlando, Luisa De Angelis, Gianluca D’Egidio, Armando Di Ludovico, Luciana Breda

**Affiliations:** 1 Pediatric Rheumatology Unit, Department of Pediatrics, SS Annunziata Hospital, Via dei Vestini, 66100 Chieti, Italy; lisa.deangelis2@gmail.com (L.D.A.); gianlucadegidio@gmail.com (G.D.); armandodl@outlook.com (A.D.L.); 2 Gabriele D’Annunzio University, Via dei Vestini, 31, 66013 Chieti, Italy

**Keywords:** group A streptococcus, autoimmune, molecular mimicry, acute rheumatic fever, rheumatic heart disease, acute post-streptococcal glomerulonephritis, guttate psoriasis

## Abstract

Group A β-hemolytic Streptococcus (GAS) is a Gram-positive, coccoid-shaped bacterium that tends to grow in chains; it is a non-spore-forming, facultatively anaerobic, catalase-negative, aerobic bacterium. It is known to cause a wide range of infections in children, ranging from mild upper respiratory tract infections, such as pharyngitis, to severe invasive disease. GAS also notably triggers post-infectious immune sequelae, including acute poststreptococcal glomerulonephritis (APSGN), acute rheumatic fever (ARF), and rheumatic heart disease (RHD), which are major health burdens, especially in low-income countries. In this review, we will present the general characteristics of GAS, highlighting its structural and microbiological features. We will also discuss its pathogenetic mechanisms, especially molecular mimicry, and its ability to cause autoimmune responses. Finally, we will elucidate some of the autoimmune sequelae that mark GAS infections, such as ARF, RHD, APSGN, and guttate psoriasis. Understanding GAS as a model pathogen for infection-induced autoimmunity provides insight into host–pathogen interactions and supports the development of targeted interventions. Emphasis on early diagnosis and antibiotic treatment is essential to reduce the burden of autoimmune complications

## 1. Introduction

Group A β-hemolytic Streptococcus (GAS) is a coccoid-shaped bacteria known to cause a wide range of infections in children, ranging from mild upper respiratory tract infections, such as pharyngitis, to severe invasive diseases, including, puerperal sepsis, bacteremia, streptococcal toxic shock syndrome (STSS), necrotizing fasciitis, endocarditis, and pneumonia. Post-infectious immune sequelae, including acute poststreptococcal glomerulonephritis, rheumatic fever, and rheumatic heart disease are a major health burden, especially in low-income countries where these complications are responsible for an estimated 600,000 annual deaths [1,2].

In this review, we will present the general characteristics of GAS, highlighting its structural and microbiological features. We will also discuss its pathogenetic mechanisms, especially molecular mimicry, and its ability to cause autoimmune responses. Finally, we will elucidate some of the autoimmune sequelae that mark GAS infections, such as ARF, RHD, APSGN, and guttate psoriasis.

## 2. Streptococcus Pyogenes

GAS, also known as *Streptococcus pyogenes* (*S. pyogenes*), is a Gram-positive, coccoid-shaped bacterium that tends to grow in chains; it is a non-spore-forming, facultatively anaerobic, catalase-negative, aerobic bacterium. The term “β-hemolytic” derives from its ability to cause complete lysis of red blood cells in culture media, a feature seen as a clear area around colonies (beta hemolysis).

GAS is characterized by the presence, in the cell wall, of the group A polysaccharide antigen (an oligomer of N-Acetylglucosamine and rhamnose).

**Figure 1:**
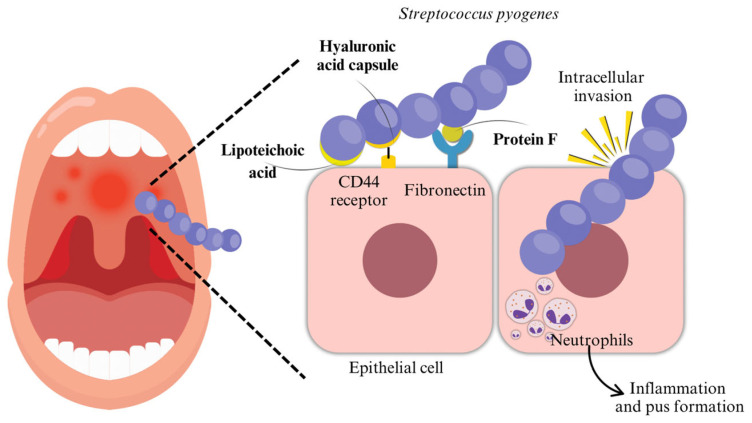
Initial infection and colonization: GAS adheres to epithelial cells through fibronectin. This interaction seems to be mediated by protein F, considered the main adhesin of the bacterium.

Humans, particularly children, are commonly transiently colonized without overt symptoms. The mechanism through which GAS adheres to epithelial cells is fundamentally represented by the interaction of the bacterial surface with the fibronectin present in the intercellular matrix. This interaction appears to be mediated by a surface protein of nearly 120 kDa, called protein F, which is to be considered the main adhesin of the bacterium (Figure 1).

### 2.1. Pathogenesis

**Figure 2:**
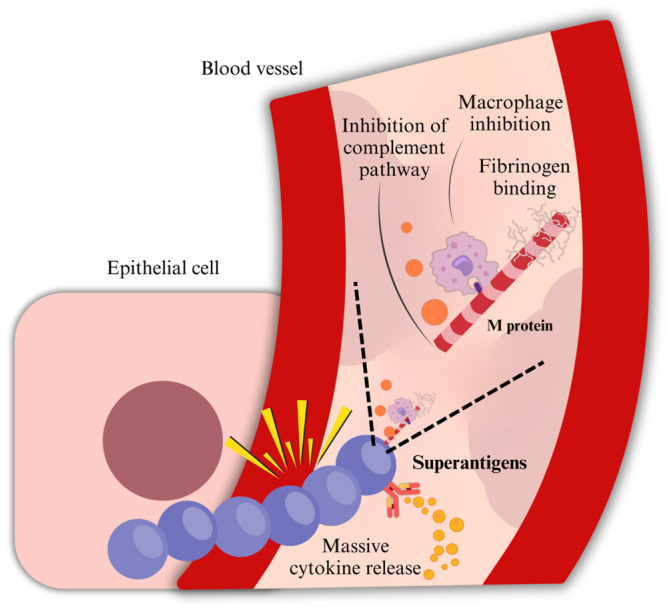
Immune evasion and systemic spread. M protein is a fibrillar protein complexed with teichoic acids, such as lipoteichoic acid (LTA), and is crucial in the pathogenicity of the bacterium. It anchors to the cell membrane with the LPSTGE motif and interferes with opsonization, inhibiting the complement pathway by binding Factor H and fibrinogen, reducing the amount of C3b bound to streptococci. Moreover, GAS produces superantigenic molecules, which enhance its virulence, playing a critical role in severe invasive infections. Immune evasion is also fostered by superantigens, highly stimulating T-CD4+ and T-CD8+ cells, resulting in an uncontrolled stimulation of T-cells and, therefore, a massive release of proinflammatory cytokines. Furthermore, GAS evades phagocytosis by producing an NADase.

The ability of GAS to cause disease is attributed to numerous virulence factors that allow it to evade the host’s immune system and cause tissue damage (Figure 2).

#### 2.1.1. Adhesion

The adhesion between streptococcal ligands to specific receptors on epithelial cells is a crucial step in GAS infection: without solid interactions, group A streptococci could not bind to host tissues and would be eliminated by mucous and salivary fluid flow processes, as well as epithelial exfoliation. The adhesion process is mediated by multiple adhesion molecules: more than 10 molecules have been described, including M protein, protein F/Sfb, a 29/kDa fibronectin-binding protein, glyceraldehyde-3-phosphate dehydroge- nase, a 70 kDa galactose-binding protein, a vitronectin-binding protein, a collagen-binding protein, serum opacity factor, a 54 kDa fibronectin-binding protein, FBP54, and the hyaluronate capsule. Moreover, the adhesion process is also mediated by several extracellular host–cell proteins, such as fibronectin, fibrinogen, and collagen [3].

The adhesion process consists of an initial weak interaction with the mucosa, followed by a second adherence event that confers tissue specificity and high-avidity adherence. The first step is reversible with multiple washing and is most likely mediated by hydrophobic interactions, such as lipoteichoic acid (LTA), which accounts for approximately 60% of adhesion to epithelial cells; the second phase involves adhesins [3,4,5,6,7,8,9,10,11,12], which are specialized for receptors expressed by cells in specific habitats, and produces a solid, practically irreversible adhesion.

#### 2.1.2. Invasion

In addition to surface adherence, GAS can actively invade host epithelial cells, which plays a significant role in its pathogenesis. This invasion creates an intracellular reservoir that may lead to persistent or recurrent infections, such as pharyngotonsillitis. Moreover, by residing within host cells, GAS can evade immune responses and antibiotic treatment, contributing to chronic carriage and therapeutic failure. However, the overall impact of GAS internalization by pharyngeal keratinocytes on bacterial persistence in the human host is thought to rely on multiple variables that determine the efficacy of internalization and intracellular death [12,13,14,15].

#### 2.1.3. Hyaluronic Acid Capsule

The hyaluronic acid capsule plays an essential role in adhesion, since it binds CD44 on pharyngeal epithelial cells [12], as well as contributing to GAS antiphagocytic activity. The capsule, made of repeating units of glucuronic acid and N-acetylglucosamine, acts as a physical barrier, preventing the access of phagocytes: as a matter of fact, data show that hyaluronidase treatment of encapsulated streptococci enhances their sensitivity to phagocytosis [16]. N-acetyl-glucosamine is the immunodominant epitope of the group A carbohydrate that distinguishes *S. pyogenes* from other streptococcal species.

#### 2.1.4. Opsonization

Moreover, GAS interferes with opsonization through one of its major virulence factors: the M protein. In the absence of M type-specific antibodies, opsonization proceeds via the alternative complement pathway, leading to the deposition of C3 and C3b fragments on the bacterial surface. These fragments are then recognized by phagocytic cells through their C3 receptors. Factor H, a key regulator of the alternative pathway, downregulates complement activation by promoting the degradation of soluble C3. Moreover, Factor I and Factor H facilitate the degradation of C3b deposited on the cell surface, further limiting opsonization. The M protein contributes to immune evasion by binding Factor H and fibrinogen, thereby inhibiting complement activity and reducing the amount of C3b deposited on the surface of streptococci [17].

#### 2.1.5. M Protein

On the GAS surface, there are some helicoidal structures composed of a fibrillar protein complexed with teichoic acids, such as lipoteichoic acid (LTA), which is crucial in the pathogenicity of the bacterium, M protein. M protein is a major surface protein and virulence factor of group A streptococci with approximately 100 serotypes identified. M1 is one of the most studied serotypes: it induces an excessive immune response, and it is frequently associated with serious invasive infections. M3 strains are known for their ability to invade human endothelial cells and are also associated with invasive infections. M28 is also common among invasive and pharyngeal infections; in addition, it is also significantly more represented among puerperal and neonatal GAS infections. M12 serotype has been identified as one of the most prevalent among non-invasive pharyngeal isolates. Other serotypes, such as M5, M6, M18, M24, and M29, have been associated with invasive infections, such as pneumonia with pleural effusion, with a greater need for pediatric intensive care unit admission than other serotypes [18,19].

M protein anchors to the cell wall through the LPXGT motif and extends from the surface of the streptococcal wall (N-terminal region) to inside the cell wall (C-terminal region) [20]. The LPXGT motif, located near the C-terminus, is recognized by an enzyme known as sortase A, which cleaves the protein precisely between the threonine (T) and glycine (G) residues. Following this cleavage, sortase A forms a covalent bond between the newly exposed threonine and the peptidoglycan precursor, lipid II, which is part of the growing bacterial cell wall [21]. 

The distal portion of M protein presents the characteristics of hypervariability to which the different serotypes correspond. M proteins share certain epitopes with numerous human fibrillar proteins, which provides a theoretical basis for the presence of (auto)antibodies that are cross-reactive with host tissues in the autoimmune sequelae of acute streptococcal infection, in genetically predisposed subjects. The M protein also exhibits potent anti-phagocytic activity, which is mediated in part by its ability to bind host regulatory proteins, such as Factor H and fibrinogen. By recruiting Factor H to the bacterial surface, the M protein promotes the degradation of C3b through Factor H’s cooperation with Factor I. This prevents effective opsonization, as C3b is inactivated and unable to facilitate recognition by phagocytic cells. Additionally, fibrinogen binding helps shield the bacterial surface, further interfering with complement deposition and phagocyte recognition [3].

M proteins are classified into two major groups: class I molecules, which react with antibodies directed against the conserved C-repeat region, and class II molecules, which do not elicit such reactivity [22].

#### 2.1.6. Exotoxins and Superantigens

A key exotoxin secreted by *S. pyogenes* is Streptolysin-O, a thiol-activated cytolysin (or hemolysin) belonging to the family of cholesterol-dependent cytolysins (CDCs). Its activity relies on the presence of sulfhydryl (-SH) groups and is inhibited in the presence of oxygen, which is why it is referred to as an oxygen-labile hemolysin (hence the name oxygen-labile hemolysins or hemolysins O). SLO exerts its effect by binding to cholesterol in the host cell membrane, forming large transmembrane pores that disrupt membrane integrity. This leads to ionic imbalance, cell lysis, and ultimately cell death, often through apoptosis. Due to its strong and consistent ability to elicit an immune response, SLO serves as a diagnostic marker: the detection of anti-streptolysin O (ASO) antibodies in patient serum is commonly used in serological tests to assess recent or past *S. pyogenes* infections [18].

Over 95% of *S. pyogenes* strains also produce another substance with a cytolytic action, namely Streptolysin-S, so called because it was initially identified only in cultures in the presence of serum. Unlike Streptolysin O, SLS is stable in the presence of oxygen and is primarily responsible for the halo of complete hemolysis observed around *S. pyogenes* colonies grown on blood agar plates under aerobic conditions. The aerobic incubation ensures that Streptolysin O is inactivated, highlighting the hemolytic activity of SLS [18]. The active form is probably represented by a dimer of the toxic peptide, and its hemolytic action seems to result from the formation of “pores” in the phospholipids of the membranes. The cytotoxicity of steptolysin-S is very high. A singular characteristic of streptolysin-S is represented by the poor or absent immunogenicity and the consequent lack of antibody response in infected subjects.

Group A streptococci can be further classified into two groups based on the presence or absence of a serum opacity factor, a lipoproteinase responsible for inducing opacity in serum [23,24,25]. The opacity factor-negative group contains the “rheumatogenic” strains, while the opacity factor-positive group contains the “nephritogenic” strains [3].

Moreover, GAS produces superantigenic molecules, which enhance its virulence, playing a critical role in toxic shock. “Superantigens” are non-glycosylated low-molecular-weight exoproteins that stimulate a large proportion of T lymphocytes by simultaneously binding to the MHC class II molecules on antigen-presenting cells and to the variable region of the β-chain (Vβ) of the T cell receptor, independently of antigen specificity. This atypical activation bypasses normal antigen processing and leads to a massive, uncontrolled release of cytokines. While antigen-presenting cells release cytokines such as IL-1 and TNF-α, activated T cells predominantly secrete IL-2 and IFN-γ, contributing to systemic inflammation, fever, and in extreme cases, toxic shock [18].

Superantigens are generally resistant to proteolysis, acids, and desiccation (toxic shock syndrome toxin-1) [26]. Among the first *S. pyogenes* superantigens identified were streptococcal pyrogenic exotoxins A, also known as erythrogenic toxin, and C (SpeA and SpeC), both implicated in scarlet fever and streptococcal toxic shock syndrome [27,28]. These toxins, like other GAS superantigens, are structurally conserved and exhibit potent pyrogenic and immunostimulatory activity. Other superantigens such as streptococcal superantigen (SSA), streptococcal mitogenic exotoxin Z (SMEZ), and a broader set from SpeG to SpeM, were discovered through genomic sequencing and share conserved family signature motifslker [27].

#### 2.1.7. Exoenzymes

In acute, the pathogenic activity of *S. pyogenes* is further facilitated by the production of several exoenzymes, primarily streptokinase (also known as fibrolysin) and hyaluronidase. Streptokinase acts on human plasminogen by catalyzing its conversion into plasmin, an enzyme capable of dissolving fibrin clots. This process enables the bacteria to evade containment mechanisms within the host. Hyaluronidase, with a similar function to the homologous enzyme found in *Staphylococcus* species, facilitates bacterial diffusion by degrading hyaluronic acid, thereby promoting the spread of *S. pyogenes* to the surrounding tissues from the primary colonization site [18,19].

Additionally, GAS expresses a C5a-peptidase (serine protease) bound to the bacterial cell surface. This enzyme degrades the complement product C5a, thus neutralizing the chemotactic signal that would otherwise attract polymorphonuclear phagocytes. By inhibiting this immune response, *S. pyogenes* effectively evades one of the body’s primary mechanisms for combating bacterial infection [18,19].

SpeB is as a cysteine protease. It contributes to tissue damage by degrading key host proteins such as fibronectin, vitronectin, and the precursor of IL-1β, and is strongly implicated in the pathogenesis of severe invasive diseases such as necrotizing fasciitis [18,19].

Furthermore, GAS evades phagocytosis by producing NADase (nicotinamide adenine dinucleotide glycohydrolase), an enzyme that acts in synergy with Streptolysin O (SLO). Together, these toxins contribute to the death of phagocytic cells. The depletion of the intracellular NAD pool impairs the leukocytes that have phagocytosed the bacterium, rendering them ineffective [18]. Additionally, GAS produces multiple DNases (mostly secreted forms and one cell wall-anchored form). These enzymes degrade extracellular DNA, which contributes not only to the formation of abscesses but also to the breakdown of neutrophil extracellular traps (NETs), a key innate immune defense mechanism. By disrupting these DNA-based structures, GAS facilitates its own dissemination through host tissues [18].

Lastly, many strains of GAS produce neuraminidase (sialidase), an enzyme that depolymerizes the mucous secretions present on the epithelia of the upper respiratory tract. This enzymatic action likely serves as a factor promoting bacterial colonization of the epithelial surfaces, aiding in the establishment of infection, which is why neuroaminidase is important in GAS virulence [18,19]. Serotypes M1, M3, and M12 are among the most studied ones and are frequently associated with neuraminidase production; however, other serotypes, such as M28, M5, M6, M18, and M24, are also relevant. Variability in neuraminidase production between different serotypes contributes to the clinical diversity of *S. pyogenes* infections [18,19].

### 2.2. Molecular Mimicry

Group A streptococci can also lead to autoimmune sequelae, which arise when cross-reactive antigens, located on the bacteria, mimic host molecules and induce an autoimmune response against host tissues. This immunological cross-reactivity is referred to as “molecular mimicry” and was first found in association to GAS [29,30]. Molecular mimicry was first defined as identical amino acid sequences shared between different molecules present in tissues and the bacterium. Subsequently, two additional types of molecular mimicry were identified, involving cross-reactivity between similar molecular structures, such as alpha-helical coiled-coil molecules (e.g., M protein and myosin) but also similar sequences shared between different types of molecules (e.g., carbohydrates and peptides) [31,32,33].

Research on the cross-reactive antigens of group A streptococci has significantly contributed to our understanding of molecular mimicry and autoimmunity. Cross-reactive antibodies and antigens were first found when rheumatic fever sera or anti-group A streptococcal antisera reacted with human heart or skeletal muscle tissues. Antibodies against group A streptococci in the serum of rheumatic fever patients were absorbed from the sera with human heart extracts and anti-heart antibodies were absorbed from the sera with group A streptococci or streptococcal membranes [34,35,36,37,38,39].

Subsequent evidence suggested that cross-reactive antigens were located in both the cell wall and membrane of the group A streptococci and that group A polysaccharide was a cross-reactive antigen [40,41,42,43,44,45]. Later, studies suggested the terminal O-linked N-acetyl-glucosamine and the hyaluronic acid capsule as cross-reactive antigens [33,46]. The M protein is also one of the cross-reactive antigens most well investigated: the immunologic similarity with cardiac myosin is supported by the presence of a seven-amino-acid-residue periodicity shared with tropomyosin, myosin, desmin, vimentin, and keratin [47,48,49]. In addition, a 60 kDa protein, located in the cell membrane, and a 67 kDa protein can be included among the GAS cross-reactive antigens: the 60 kDa wall-membrane antigen reacts with cardiac myosin, while the 67 kDa protein mimics the class II MHC molecules [50,51].

Studies on molecular mimicry and GAS led to the identification of three main subset of cross-reactive antibodies, based on their cross-reactivity with DNA, N-acetyl-glucosamine or myosin and other alpha-helical molecules (e.g., tropomyosin, vimentin, laminin). In humans, the predominant subset appears to react with the N-acetyl-glucosamine epitope, as well as with myosin and related molecules. This finding is not surprising, as patients with rheumatic fever do not develop antinuclear antibodies during the disease.

The development of T lymphocytes recognizing myosin and other self-proteins homologous to M protein explains the onset of rheumatic myocarditis but does not fully account for the involvement of heart valves, which is the primary contributor to the disease’s morbidity. Valvular pathology seems to stem from anti-laminin T lymphocytes, partially cross-reactive with M protein, and, at least in part, from the production of autoantibodies directed against N-acetyl-glucosamine in group A carbohydrates, which cross-react with valvular tissue. Cross-reactive antibodies primarily target the valve endothelium and laminin and start the endocardial inflammation. The expression of Vascular cell adhesion molecule-1 (VCAM-1) attracts activated T cell through VLA-4, enhancing the inflammation and leading to eventual scarring and neovascularization of the normally avascular valve [52]. Furthermore, the extracellular matrix may act as a filter, retaining cross-reactive antibodies.

As a result, Aschoff bodies develop beneath the endocardium, progressively damaging the heart valves: these nodules are granulomatous lesions comprising a central area of fibrinoid necrosis, which is encircled by specialized histiocytes, including Aschoff giant cells and Anitschkow cells (caterpillar cells) and lymphocytes [42]. The earliest lesions contain edema, lymphocytes, histiocytes, and plasma cells; subsequently, the accumulation of characteristic Aschoff giant cells and Anitschkow cells mark the specific granulomatous stage, followed by the late stage with diminution of the cellular infiltrate and replacement by scar tissue [53,54,55].

Lastly, the cross-reactivity between GAS and laminin, type IV collagen, and other macromolecules found in the glomerular basement membrane explains the acute post-streptococcal glomerulonephritis [56]. As a matter of fact, studies found streptococcal antigens, immunoglobulins, and complements in the glomeruli of patients with APSGN. Other research suggested that the glomerular basement membrane shares antigens with streptococcal M12 protein [57], while a renal autoimmune epitope was identified in the M protein [58].

## 3. Group a Streptococcus-Associated Autoimmune Sequelae

**Figure 3:**
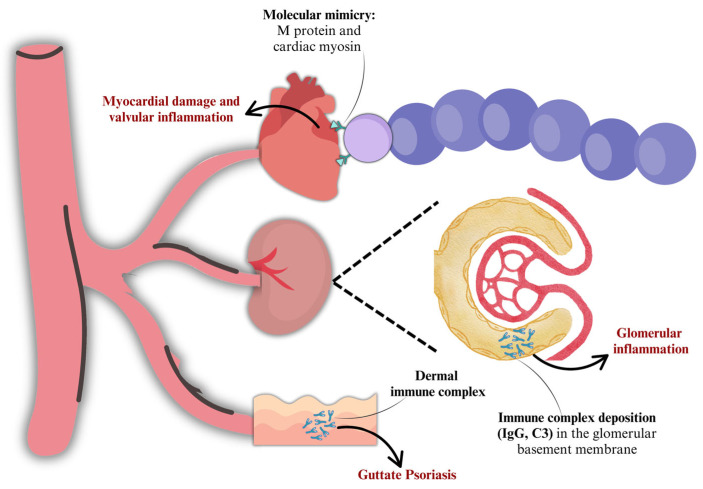
Post-infectious and autoimmune sequelae.

Autoimmune sequelae can be known from Figure 3.

### 3.1. Acute Rheumatic Fever (ARF) and Rheumatic Heart Disease (RHD)

Acute rheumatic fever and rheumatic heart disease have progressively declined over the last hundred years in all high-income countries, as a result of improved quality of life, greater access to medical care, and the consequent reduction in streptococcal infections. In contrast, the disease remains prevalent in developing countries, where it is the leading cause of heart disease in children and a significant source of morbidity and mortality in adults.

The first episodes of ARF mostly occur in children aged 5–14 years; although, there are reported cases of ARF in 2–3 year-old-children. Cases of ARF in individuals over the age of 30 are exceedingly rare. Conversely, the prevalence of RHD is highest in adulthood, typically between the ages of 25 and 45 [59]. Approximately 3% of individuals with untreated group A streptococcal infections may develop rheumatic disease. ARF is equally common in males and females; however, RHD occurs more commonly in females, probably due to greater autoimmune susceptibility [59].

Poverty and social disadvantage are among the most significant risk factors for the development of acute rheumatic fever, potentially due to transmission of GAS through domestic overcrowding. Ethnicity may serve as an additional risk factor; however, the elevated susceptibility of certain ethnic groups may be attributed to their elevated rates of poverty and overcrowding, rather than their genetic susceptibility [59].

ARF results from the interaction between a wide variety of GAS *emm* types and a genetically predisposed individual. Usually, the trigger infection is a pharyngitis; although, some studies suggest that skin infections might lead to the autoimmune sequela as well: the activation of adaptive and immune responses leads to a non-suppurative inflammatory reaction, which affects, through molecular mimicry, joints, heart, and/or central nervous system (CNS). All manifestations resolve spontaneously, except for the damage to the heart valves, which persists over time and is responsible for the significant morbidity of the disease.

Autoantibodies induce endothelial activation facilitating T-cell infiltration into the avascular valve matrix: local tissue damage is mediated through a T-helper cell 1 response, leading to inflammatory cytokines release (IFNγ, TNFα) and a reduction in IL-4 and IL-10. The immune damage is further amplified by recognition of other structural valve proteins such as vimentin and collagen through local epitope spreading. However, antibodies against collagen do not induce valvulitis in animal models. Therefore, molecular mimicry is probably essential for induction of autoimmunity and initiation of valve damage during acute rheumatic fever, and antibodies against collagen could contribute to disease progression. Additionally, Th17 responses occur with group A streptococcal infections, and patients with acute rheumatic fever and rheumatic heart disease have increased numbers of Th17 cells and higher IL17 concentrations [60,61].

#### 3.1.1. Clinical Presentation

Fever occurs in more than 90% of patients: a low cut-off (38 °C) can help diagnose ARF in endemic areas [59,60]. Arthritis is the second most common clinical feature (75% of first episodes of ARF) and usually presents with an initial monoarthritis, followed by a migratory polyarthritis, mostly involving large joints (knees, ankles, elbows, and wrists). Polyarthralgia or monoarthralgia can also be seen. Joint manifestations are highly responsive to anti-inflammatory drugs; although, they can be self-limiting and can resolve with or without treatment within 4 weeks [59,60].

Carditis is the most serious presentation of ARF: heart involvement occurs in 50% to 80% of patients with ARF and usually presents as pancarditis within 2 to 3 weeks, involving the pericardium, myocardium, and endocardium. Pericardium involvement manifests as a pericarditis, which resolves without sequelae. Myocardium typically is not affected in its entirety but rather in specific regions, and as a result, its involvement may not lead to systolic dysfunction. Endocardial involvement in the form of valvulitis presents as regurgitation of the mitral valve (50–60%) and, less commonly, of the aortic valve (20%). The tricuspid valve is only affected in 10% of cases of valvulitis and typically results in tricuspid regurgitation. Clinical manifestations can include palpitations, dyspnea, and heart failure (when regurgitation is severe due to leaflet prolapse or chordal rupture); however, mitral regurgitation is usually the earliest manifestation. Carditis is diagnosed clinically in 50–70% of cases; subclinical carditis is diagnosed in an additional 12–21% of cases [60,62].

Progressive valvular damage can lead to valvular stenosis. The transition from ARF to RHD occurs when valvular lesions evolve over the years or during multiple episodes of ARF. However, heart failure can occur, even during acute rheumatic fever, due to severe pancarditis or valvulopathy.

Dermatological manifestations in ARF include erythema marginatum, a patognomonic feature, which consists of non-pruritic, bright pink, blanching macules or papules that spread outwards in a circular pattern with serpiginous borders. It usually appears on the trunk and proximal limbs and is transient, fading and reappearing elsewhere [60,63]. Subcutaneous nodules are small (0.5–2 cm diameter) and painless round nodules that develop over bony prominences or extensor surfaces of the limbs and along the spinous processes of the vertebrae. They occur in less than 10% of patients with ARF and are typically associated with severe carditis [59,60].

Sydenham Chorea is another major manifestation of ARF and can be seen in 10–30% of cases. It is characterized by involuntary, purposeless movements of the trunk, limbs, and face. Cardiac involvement is common in patients with chorea, especially considering subclinical involvement (up to 90% of patients). Chorea usually appears 1–3 months after an ARF episode and can also occur as an isolated manifestation [59,60,64]. Molecular mimicry induces the production of antibodies targeting the CNS: studies showed specificity for lysoganglioside, N-acetyl-β-D-glucosamine, and tubulin; however, the most interesting finding was the interaction between chorea IgGs and dopamine receptors D1 and, more importantly, D2 [33,65]. This identification led to the definition of Sydenham Chorea as a dopamine receptor encephalitis. After an ARF episode, chorea antibodies penetrate the brain blood barrier and reach brain tissues, more specifically dopaminergic neurons in the ventral tegumental area or the substantia nigra: the interaction with D1 and D2 on dopaminergic neurons induces an elevated activity of calcium/calmodulin-dependent protein kinase II (CaM kinase II), which then leads to the production and release of excess dopamine by neuronal cells [33,65,66]. Similar autoantibodies are elevated in serum and CSF from patients with PANDAS with the choreiform movements. Moreover, other animal models demonstrated that cross-reactive antibodies induced neuropsychiatric symptoms and typical OCD behavior [67].

#### 3.1.2. Diagnosis

No single laboratory test or clinical feature is diagnostic of acute rheumatic fever: throat swabs are usually negative for GAS, and their positivity should not be considered since throat colonization can take place. Laboratory tests such as anti-Streptolysin O (ASO) and anti-DNAse B can be more helpful to establish a preceding GAS infection.

The Jones Criteria, since 1944, have represented the clinical standard to establish the diagnosis of ARF. However, these criteria have been modified many times by the American Heart Association; the last one goes back to 2015, identifying major and minor criteria in high-risk populations (Table 1) and low-risk populations. In this revision, subclinical carditis was added as a major criteria (Table 2) [68]. A documented past infection and/or a precedent diagnosis of ARF are necessary for a correct diagnosis. In Table 3, we report the criteria combination established in the revised Jones Criteria [68].

A documented past infection and/or a precedent diagnosis of ARF are necessary for a correct diagnosis. In Table 3, we report the criteria combination established in the revised Jones Criteria [68].

Differential diagnosis can be a challenge, especially regarding arthritis: in patients with a single inflamed joint, it is important to exclude septic arthritis; moreover, as stated above, ARF arthritis is highly responsive to anti-inflammatory drugs, which is why in case of a lack of clinical response within 48–72 h, an alternate diagnoses should be considered. Cardiac involvement must be assessed: echocardiographic evaluation can be a great advantage, especially in case of subclinical carditis. Moreover, the severity of the carditis is of great importance in terms of prognostic evaluation, follow-up, and secondary prophylaxis. If chorea is present, it is important to exclude other potential causes, including drug reactions, systemic lupus erythematosus, Wilson disease, and other diseases, and to carry out an echocardiogram because chorea is strongly associated with carditis.

#### 3.1.3. Treatment and Primary Prevention

One main aim of the treatment of ARF is the eradication of GAS: it can be achieved either with penicillin, a single dose of intramuscular benzathine benzylpenicillin, or a 10-day course of oral phenoxymethylpenicillin or oral amoxicillin. In case of allergies, azithromycin or clarithromycin should be used [69]. In Table 4, we report primary prevention strategies.

Joint manifestations and symptoms can be quickly controlled with Aspirin or other NSAIDs: the response occurs within 48–72 h to treatment with high-dose aspirin (80–100 mg/kg per day in three or four divided doses). Treatment is usually required for 1–4 weeks but can be given for up to 12 weeks. Aspirin commonly causes gastrointestinal side-effects, and proton pump inhibitors are often co-prescribed. Naproxen (15–20 mg/kg/day in 2 divided doses) and ibuprofen (30–40 mg/kg/day in 3–4 doses) can be valid alternatives [59,60].

Angiotensin-converting enzyme inhibitors are recommended for carditis, together with symptomatic treatment with diuretics. Corticosteroids decrease inflammatory markers associated with acute rheumatic fever, particularly fever and elevated acute phase reactants. They are commonly employed in cases of severe acute carditis accompanied by heart failure, despite a lack of substantial objective evidence supporting their efficacy in enhancing standard treatments, including bed rest, fluid restriction, and cardiac medications. In patients with heart failure, diuretics and fluid restriction are pillars of the treatment strategy. More severe scenarios might need cardiac surgery, such as chordae tendinae rupture. Cardiac surgery in young patients aims to the replacement of the mitral valve [59,60,70].

Chorea usually resolves spontaneously, but symptomatic treatment, such as dopamine antagonists, might be administrated; however, they are usually associated with extrapyramidal side-effects that limit patients’ daily activities. Antiepileptic drugs, carbamazepine and sodium valproate, provide symptomatic relief with fewer side-effects and might be preferable over dopamine antagonists.

#### 3.1.4. Secondary Prevention

The prevention of progression from acute rheumatic fever to rheumatic heart disease is crucial: benzathine penicillin G is the gold standard of treatment for its long-acting effects and should be administered every 21 days intramuscularly. Oral agents are more appropriate for patients at lower risk for rheumatic fever recurrence. Accordingly, some physicians may consider switching patients to oral prophylaxis when they have reached late adolescence or young adulthood and have remained free of rheumatic attacks for at least 5 years. The recommended oral agent is penicillin V 250 mg twice daily.

The duration of secondary prevention varies depending on the severity of ARF episodes: in case of severe carditis with residual heart disease (a), treatment should be continued for at least 10 years or until 40 years of age (whichever is longer); after that time, the severity of the valvular disease and the potential for exposure to GAS should be discussed, and continued prophylaxis (potentially lifelong) should be considered for high-risk patients. If the patient presented ARF with carditis but no residual heart disease, (b) treatment should be continued for 10 years or until 21 years of age (whichever is longer). In case of patients with ARF but without carditis, secondary prevention should be continued for at least 5 years or until 21 years of age (whichever is longer) [69]. In Table 5, we summarize secondary prophylaxis strategies.

In case of allergies to penicillin, sulfadiazine is recommended: although sulfonamides are not effective in the eradication of GAS, they do prevent infection. The recommended dose of sulfadiazine is 0.5 g/day for patients weighing 27 kg or less and 1 g/day for patients weighing more than 27 kg. In patients allergic to both penicillin and sulfisoxazole, an oral macrolide is recommended, such as azithromycin.

### 3.2. Acute Post-Streptococcal Glomerulonephritis (APSGN)

Throughout most of the last century, APSGN was the prototypical bacterial infection-associated glomerulonephritis. Nevertheless, enhancements in healthcare (early identification and treatment of pharyngitis and skin infections, vaccination programs) and socioeconomic situations led to a diminished spread of infections and averted disease epidemics, resulting in a significant reduction in the incidence of APSGN [71,72]. Despite remaining the leading cause of acute nephritis in children, it can also be classified as a disease of disparities, similar to RHD, as it predominantly affects developing countries.

APSGN mostly affects pediatric patients (3–14 y.o.) and can be triggered by throat infections when associated with nephritogenic GAS strains 12 and 4, and the association with skin infections is mainly related to M types 49, 42, 2, 57, and 60 [72]. Nevertheless, GAS infection is not necessarily followed by APSGN: studies show that less than 2% of children infected with nephritogenic GAS strains develop acute GN [71].

The main theory on APSGN pathogenesis is based on glomerular trapping of circulating immune complexes as well as in situ immune complex formation resulting from molecular mimicry. Serum complement profiles and immunofluorescence studies indicate that C3 activation primarily follows the alternative pathway: immune deposits generally include IgG, C3, properdin, and C5, but rarely involve classical pathway components like C1q or C4. The presence of C5b-9 and its regulatory protein, vitronectin, aligns with C3, suggesting that terminal complement pathway activation occurs locally in the glomerulus. Early on, some patients may show classical pathway activation, with temporary decreases in C1q, C2, or C4, and circulating C1-inhibitor-C1r-C1s complexes during the first two weeks. This observation of classical complement pathway activation may indicate the presence of circulating immune complexes during the acute phase, which are separate from the immune deposits found in the glomeruli [25].

#### 3.2.1. Clinical Presentation

The majority of APSGN cases are subclinical; however, it may present as an acute nephritic syndrome with its classical triad: edema, linked to salt and water retention, hematuria, and hypertension. Except atypical presentation, hematuria occurs in almost all patients, with tea-colored or cola-colored urine seen in about 25–60% of cases. Proteinuria is common, but nephrotic syndrome is rarely observed [25].

Acute kidney injury is reported in 20% of cases; however, dialysis is uncommonly needed. Renal dysfunction can also present with hypertension: cerebral complications include headaches, seizures, mental status changes, and visual changes. Diastolic blood pressure correlates with fluid overload, which can be assessed with weight changes. Atypical features can be pulmonary edema and Henoch–Schönlein purpura rash.

Second attacks of PSAGN have been reported but are rare [25].

#### 3.2.2. Diagnosis

C3 is commonly low during the acute phase, probably owing to the activation of the complement cascade and increased consumption of complement factors due to the formation of immune complexes. However, a normal serum C3 concentration can also be seen at clinical onset. As for ARF, ASO serum titers can be helpful for the individuation of a preceding GAS infection.

Histologically, APSGN is defined by diffuse glomerular endocapillary hypercellularity, characteristic of exudative glomerulonephritis. While crescents are uncommon, they may be observed in severe cases. Electron microscopy identifies subepithelial electron-dense deposits (humps), which, although not pathognomonic, represent the most characteristic finding in APSGN, even though they can also be found in other forms of glomerulonephritis. Three distinct patterns can be observed in APSGN: the *garland pattern*, where deposits align along the glomerular capillary walls, the *starry sky pattern*, which describes randomly distributed deposits, and a third pattern characterized by predominantly *mesangial staining* [72].

#### 3.2.3. Treatment

While a renal biopsy is generally not indicated in cases of suspected APSGN, it may be warranted if the clinical condition deteriorates over time or in normocomplementemic patients.

The primary approach to treatment focuses on managing symptoms, which includes reducing daily sodium intake to 2000–2500 mg and limiting fluid intake to 2000 mL, alongside the use of diuretics to address hypertension and fluid overload. If additional blood pressure regulation is required, calcium channel blockers can be used, although ACE inhibitors are contraindicated as they may lead to a decline in kidney function and increase the risk of hyperkalemia. In severe cases, dialysis may be necessary to avoid life-threatening neurological and cardiovascular complications until the acute nephritis phase resolves [72].

There is no definite evidence on immunosuppressive therapy; however, studies showed a faster recovery of the renal function [72,73]. In severe cases of APSGN, high-dose corticosteroids, starting with pulse methylprednisolone, may be warranted to suppress acute inflammation, limit chronic renal damage, and facilitate rapid renal recovery.

### 3.3. Guttate Psoriasis

Guttate psoriasis is a chronic, inflammatory, immune-mediated skin disorder affecting 0.5–2% of children [74]. It often follows a streptococcal infection, usually after 2–4 weeks, and is characterized by the sudden appearance of multiple small, erythematous, drop-like scaly papules and plaques on the trunk and extremities.

In guttate psoriasis, the inflammation is T cell-mediated and is characterized by an imbalance between T helper 1 and T helper 2 cells, with an upregulation of Th1 activation: as a matter of fact, IL-2, IL-17, IFNγ, and TNF levels (all Th1 cytokines) are elevated. IL-2 stimulates growth of T cells, IFNγ inhibits apoptosis of keratinocytes, and TNF increases proliferation of proinflammatory cytokines and adhesion molecules. In contrast, Th2 cytokines, such as IL-10, are downregulated [74,75].

An interplay between genetic and environmental factors may contribute to disease development. A family history of psoriasis in a first-degree relative is present in approximately 30% of patients with childhood-onset psoriasis [74,76].

Guttate psoriasis typically presents with an abrupt onset of numerous, small, scattered, tear-drop-shaped (‘guttate’), erythematous papules and plaques. Lesions are usually 2–6 mm in diameter, pruritic, and primarily present on the trunk and proximal extremities. Face, scalp, hands, and feet might also be affected; however, palms and soles are usually spared. The eruption may occur de novo in individuals without a prior history of psoriasis or present as a new variant in those with pre-existing plaque psoriasis. As with all forms of psoriasis, the Koebner phenomenon is a characteristic feature [74].

The diagnosis is mainly clinical based on the characteristics of the lesions and the correlation with a previous streptococcal infection. Dermoscopy can assist in the diagnosis, typically revealing a dull-red or bright-red background, with dotted vessels arranged diffusely, along with diffuse white scales [74,77]. Laboratory tests are usually not necessary; however, in case of features of streptococcal infection, a culture from an appropriate site and measurement of serum antistreptococcal antibody titers might be helpful. Guttate psoriasis should be differentiated from other variants of psoriasis as well as pityriasis rosea, nummular eczema, and tinea corporis [77,78].

Given the potential for spontaneous resolution, active treatment may not always be necessary, except for cosmetic reasons or to manage pruritus. However, 40–50% of guttate psoriasis cases can persist and progress to chronic plaque psoriasis: factors to consider include patient age, disease severity, quality of life impact, comorbidities, prior treatment response, and patient preferences [79].

General measures include the avoidance of triggering and exacerbating factors, as well as scratching, as it may lead to the development of new psoriatic lesions (Koebner phenomenon). Topical therapies include emollients, moisturizers, and keratolytics (e.g., urea, lactic acid, dimethicone, and salicylic acid), which can help soften affected areas, together with topical corticosteroids, which remain the most rapid and effective treatment and are considered the first-line therapy for mild guttate psoriasis [74,79]. Due to the risk of side effects, the applications should be limited to no more than twice daily. Topical corticosteroids should be tapered, discontinued, or used intermittently once sufficient improvement is achieved. Topical vitamin D analogs (e.g., calcitriol and calcipotriene) promote keratinocyte differentiation and inhibit keratinocyte proliferation. Due to the risk of hypercalcemia from transcutaneous absorption, these analogs should be applied no more than once daily to less than 30% of the total body surface area. A combination of vitamin D analogs and corticosteroids is often recommended for their synergistic effects [80].

Topical calcineurin inhibitors (e.g., tacrolimus and pimecrolimus) disrupt inflammatory processes in the skin without reducing collagen synthesis or causing skin atrophy or depigmentation. Nevertheless, these agents have not been approved for psoriasis treatment, and their use for this condition is considered off-label [74,81].

UV phototherapy is the first-line treatment for moderate-to-severe guttate psoriasis, particularly when it is refractory to topical therapies or involves more than 10% of the body surface. Its efficacy, low risk of serious side effects, and ability to treat large body areas make it a preferred choice [74,80]. Narrow-band UVB (wavelength 311–313 nm) has shown superior efficacy compared to broad-band UVB (290–320 nm) and is recommended for children over six years old [82,83].

Systemic therapies are considered for moderate-to-severe guttate psoriasis when phototherapy and topical treatments fail. Methotrexate is a first-line systemic therapy, being effective, low-cost, reasonably safe, and available in an oral formulation. Methotrexate can be administered orally or subcutaneously once per week, with a recommended dose of 10–15 mg/m^2^ (not exceeding 25 mg per week) [81,84]. Folic acid supplementation is necessary to reduce the risk of deficiency. Cyclosporine is an immunosuppressant that inhibits T-cell function and reduces levels of proinflammatory cytokines. It works quickly, with improvement typically seen within 4 weeks. The recommended oral dose is 2.5–5 mg/kg/day, divided into two doses. It is often considered for children who cannot tolerate or have not responded to other systemic therapies, such as methotrexate [80]. Cyclosporine may be used alone or combined with other treatments to reduce its duration and dose; however, combining cyclosporine with phototherapy should be avoided due to an increased risk of lymphoma and skin cancer. Retinoids, like acitretin or isotretionin, are used for the treatment of guttate psoriasis. These agents do not suppress the immune system and are easy to administer (orally). The recommended dose is 0.25–1 mg/kg/day; however, they should be avoided in females of childbearing age unless contraception is used during and for 3 months after treatment due to the risk of birth defects [74].

## 4. Clinical and Therapeutic Implications

Quick identification and treatment of streptococcal infections are paramount in mitigating severe complications, particularly the transition to post-infectious autoimmune sequelae. Group A Streptococcus (GAS), a significant pathogen implicated in diseases such as acute pharyngitis, scarlet fever, and invasive streptococcal infections, is also a key etiological agent in acute rheumatic fever (ARF) and post-streptococcal glomerulonephritis (PSGN). The administration of beta-lactam antibiotics, like as penicillin, within the early stages of infection effectively reduces the incidence of immune-mediated complications [85].

Rapid antigen detection tests (RADTs) and nucleic acid amplification tests (NAATs) have revolutionized the diagnostic landscape by enabling an accurate identification of GAS infections. Combining these diagnostic modalities with clinical decision rules, such as the Centor or McIsaac criteria, ensures judicious use of antimicrobials, decreasing the emergence of antibiotic resistance while addressing the infection at its source. Chronic streptococcal carriers (defined as individuals with positive throat cultures for GAS without clinical findings or immunologic response to GAS antigens) usually do not need to be identified or treated with antibiotics [69].

### 4.1. Strategies to Prevent Progression Toward Autoimmune Diseases

Preventing the progression from acute streptococcal infections to autoimmune diseases needs integrative approaches, including vaccination, chemoprophylaxis, and public health interventions. The global burden of disease caused by GAS is not known; the prevalence of severe GAS disease is at least 18.1 million cases, with 1.78 million new cases each year. The greatest burden is due to rheumatic heart disease, especially in less developed countries [86]. Vaccines targeting M protein epitopes, as well as conserved antigens such as SpyCEP and streptolysins, represent a pivotal frontier in preventing initial GAS infections and their sequelae. Although no licensed vaccines currently exist, ongoing research demonstrates promising immunogenicity and efficacy in preclinical and early-phase trials.

In high-risk cohorts, such as individuals with a prior history of acute rheumatic fever (ARF) or rheumatic heart disease (RHD), secondary prophylaxis using intramuscular benzathine penicillin G remains the cornerstone of recurrence prevention [87]. This intervention significantly attenuates the progression of valvular damage and reduces long-term morbidity. Additionally, public health measures aimed to improving sanitation, access to healthcare, and educational initiatives are critical to reducing GAS transmission, particularly in endemic regions.

### 4.2. Potential Therapeutic Developments Targeting Pathogenic Mechanisms

Advances in understanding the immunopathogenesis of GAS infections have helped the development of novel therapeutic strategies like the progress in M protein-based vaccine formulations designed to obviate molecular mimicry and the resultant autoimmune activation while maintaining robust immunoprotection [88]. Concurrently, biological agents targeting proinflammatory cytokines, such as IL-1 and TNF-alpha, are emerging as potential adjuncts in mitigating the hyperinflammatory responses characteristic of ARF and related conditions.

Emerging modalities, such as microbiome-based interventions, aim to restore gut and mucosal microbial equilibrium, thereby modulating systemic immune responses. Regenerative medicine approaches, including stem cell therapies and bioengineered scaffolds, are being investigated for their potential to repair end-organ damage, particularly in advanced cases of RHD [89]. These innovative therapies hold promise for addressing the long-term sequelae of autoimmune pathology induced by GAS infections: they aim to prevent acute infections and promote long-term health through diverse mechanisms, including community restructuring or targeted delivery of bioactive molecules [90].

Their ecological effects can be classified into three categories: species introduction, species depletion, and enhanced growth. Applications include infection prevention and treatment by means of competitive exclusion, the production of antimicrobial compounds, or the direct depletion of pathogens. They also encompass the restoration of gut microbiota, particularly through the reintroduction of depleted microbial taxa following antibiotic therapy or dietary imbalances. Furthermore, the microbiome plays a role in the prevention and management of chronic diseases by modulating microbial contributions to host pathophysiology. Early immune system programming can be influenced by targeting the neonatal microbiome, thereby shaping both mucosal and systemic immune development. Microbial interactions also enhance nutritional outcomes through complex microbe–nutrient–host dynamics. In addition, the microbiota can improve vaccine efficacy by promoting antigenic mimicry or amplifying immunostimulatory pathways. Lastly, the composition of the microbiome may significantly alter pharmacological responses by affecting drug metabolism and overall treatment efficacy [91].

#### Antimicrobial Resistance

The global rise in antimicrobial resistance among *Streptococcus* species, particularly *S. pneumoniae* and *S. pyogenes*, highlights the urgent need for innovative therapeutic strategies. To date, GAS still maintains a high susceptibility to β-lactams, and no penicillin-resistant strains have been identified [92]. However, a reduced penicillin susceptibility has been recently reported, and the emergence of mutations in certain penicillin binding protein (PBP) genes has been considered a first step towards a potential full penicillin resistance [93,94]. The three primary mechanisms of resistance to β-lactam antibiotics are (1) enzymatic degradation of the drug by β-lactamases, (2) decreased binding affinity due to alterations in penicillin-binding proteins (PBPs), and (3) reduced access of the antibiotic to its target PBPs. In *Streptococcus pyogenes*, although β-lactamase production is rare, reduced susceptibility has been linked to mutations in PBPs. The most commonly reported alteration is a threonine-to-lysine substitution at position 553 (T553K) in the transpeptidase domain of PBP2x, which diminishes the protein’s affinity for β-lactam antibiotics such as penicillin [94,95].

Conversely, a resistance to macrolides has been described. The rates vary between 5% and 40%, with the highest prevalence in Asia and the lowest prevalence in Europe and the USA. The drastic increase in macrolide resistance can be attributed to an increased practice of azithromycin prescription for respiratory infections, which was uncommon in the previous years [96].

The main molecular mechanisms of macrolide resistance in *Streptococcus* are ribosomal modification and active antibiotic efflux. These mechanisms are mediated by distinct genetic determinants that vary in prevalence across different geographic regions. Ribosomal modification, either post- or pre-transcriptional, is primarily mediated by ***erm*** genes—such as *erm(B)*, *erm(TR)*, and *erm(T)*—which encode ribosomal methylases. These enzymes methylate the 23S rRNA component of the bacterial ribosome, reducing macrolide binding and thereby conferring high-level resistance.

The second mechanism involves the active expulsion of antibiotics through efflux pumps. This process is commonly associated with the mef(A/E)-mrs(D) gene pair, where *mef* encodes a macrolide-specific efflux transporter, and *mrs(D)* encodes a ribosomal protection protein that may enhance resistance levels. These resistance determinants are not evenly distributed worldwide. For example, *erm(B)* tends to be more prevalent in Europe and Asia, while *mef(A/E)* is more frequently observed in North America [97].

This variation reflects differences in antibiotic usage patterns, public health policies, and clonal dissemination of resistant strains. Understanding the global distribution and molecular basis of macrolide resistance is critical for designing effective treatment guidelines and surveillance strategies.

In addition, antimicrobial peptides (AMPs) have emerged as promising agents, offering novel mechanisms and reduced resistance potential compared to conventional antibiotics. AMPs are typically short, amphipathic, and cationic peptides capable of rapidly disrupting bacterial membranes, leading to cell death through mechanisms that are largely independent of classical metabolic targets. This mechanism lowers the risk of resistance selection, a key asset against multidrug-resistant organisms [98]. In addition to their direct antimicrobial effects, many AMPs also exhibit immunomodulatory properties, further enhancing their therapeutic potential [98,99]. Natural and synthetic AMPs may constitute a promising therapeutic class against *Streptococcus* spp., combining broad-spectrum efficacy with a low propensity for resistance.

## 5. Conclusions

*S. pyogenes* represent a paradigm for understanding the interplay between microbial pathogens and host autoimmunity. Its well-documented ability to trigger autoimmune conditions such as ARF and PSGN underscores its significance in elucidating the mechanisms of molecular mimicry, immune dysregulation, and host–pathogen interactions. Insights acquired from GAS research extend to broader paradigms of infection-driven autoimmunity, as a model organism in this domain [3].

Further research of the immunopathogenic mechanisms underlying GAS infections and their progression to autoimmunity is imperative. The literature highlights the critical need for advanced vaccine platforms that address the global burden of GAS-associated diseases [88]. Precision immunotherapy, tailored to the individual’s immunogenetic landscape, offers potential for mitigating autoimmune progression in susceptible populations.

Moreover, targeting superantigens might be an interesting strategy for vaccines development: evidence shows that passive immunization with anti-SpeA antibodies or vaccination with a SpeA toxoid lacking superantigenic activity confers protection against nasopharyngeal infection, while active immunization with SAgs or related toxins like staphylococcal enterotoxin B (SEB) induces protection even in the absence of specific anti-SpeA antibodies [100]. However, the variable M-protein-based vaccines face challenges due to antigenic variability and potential autoimmune reactions have complicated vaccine development [101].

In conclusion, interest in translational and clinical research, coupled with global collaborative efforts, is essential to drive innovation in diagnostic, therapeutic, and preventive strategies. By influence interdisciplinary methodologies, the scientific community is poised to develop transformative interventions to combat the far-reaching impact of streptococcal infections and their autoimmune sequelae.

## Figures and Tables

**Table 1:** Revised Jones Criteria [68]: high-risk population.

B. Major Criteria	C. Minor Criteria
Clinical and/or subclinical carditis ^1^	Monoarthralgia
Mono-/polyarthritis	Fever (≥38 °C)
Polyarthralgia	ESR ≥ 30 mm/h and/or CRP ≥ 3.0 mg/dL
Sydenham Corea	Prolonged PR interval
Erythema marginatum	
Subcutaneous nodules	

^1^ Subclinical carditis defined as echocardiographic valvulitis. ESR: Erythrocyte Sedimentation Rate. CRP: C-Reactive Protein.

**Table 2:** Revised Jones Criteria [68]: low-risk population ^2^.

B. Major Criteria	C. Minor Criteria
Clinical and/or subclinical carditis	Polyarthralgia
Polyarthritis	Fever (≥38.5 °C)
Sydenham Corea	ESR ≥ 60 mm/h and/or CRP ≥ 3.0 mg/dL
Erythema marginatum	Prolonged PR interval
Subcutaneous nodules	

^2^ Low-risk population: ARF incidence ≤ 2 per 100,000 school-aged children or all-age rheumatic heart disease prevalence of ≤ 1 per 1000 population per year. ESR: Erythrocyte Sedimentation Rate. CRP: C-Reactive Protein.

**Table 3:** Revised Jones Criteria [68]: ARF diagnosis.

Preceding GAS Infection	Criteria
Diagnosis of initial ARF	2 major criteria 1 major criteria + 2 minor criteria
Diagnosis of recurrent ARF	2 major criteria 1 major criteria + 2 minor criteria 3 minor criteria

**Table 4:** Primary prevention of acute rheumatic fever: GAS eradication [69].

Agent	Dose	Duration
Benzathine penicillin G	≤27 kg: 600,000 U i.m. >27 kg: 1,200,000 U i.m.	Single dose
Phenoxymethyl penicillin (Penicillin V)	≤27 kg: 250 mg (2–3 times/day orally) >27 kg: 500 mg (2–3 times/day orally)	10 days
Amoxicillin	50 mg/kg daily orally (max. 1 g)	10 days
Azithromycin	12 mg/kg/day (max. 500 mg)	5 days
Clarithromycin	15 mg/kg/day in 2 doses (max. 250 mg BID)	10 days
Clindamycin	20 mg/kg/day in 3 doses (max. 1.8 g/d)	10 days

**Table 5:** Secondary prevention.

Clinical Presentation	Duration
ARF with carditis and residual heart disease (persistent valvular disease ^3^)	10 years or until 40 years of age (longer option), sometimes lifelong.
ARF with carditis but no residual heart disease (no valvular disease)	10 years or until 21 years of age (longer option)
ARF without carditis	5 years or until 21 years of age (longer option)

^3^ Persistent valvular disease: clinical and/or echocardiographic.

## Data Availability

No new data were created or analyzed in this study.
